# The role of culture–gene coevolution in morality judgment: examining the interplay between tightness–looseness and allelic variation of the serotonin transporter gene

**DOI:** 10.1007/s40167-013-0009-x

**Published:** 2013-08-29

**Authors:** Alissa J. Mrazek, Joan Y. Chiao, Katherine D. Blizinsky, Janetta Lun, Michele J. Gelfand

**Affiliations:** 1 Department of Psychology, Northwestern University, Swift Hall, 2029 Sheridan Rd., Evanston, IL 60208 USA; 2 Interdepartmental Neuroscience Program, Northwestern University, Chicago, IL 60611 USA; 3 Department of Psychiatry and Behavioral Sciences, Northwestern University, Chicago, IL 60611 USA; 4 Department of Psychology, University of Maryland, College Park, MD 20742 USA

**Keywords:** Culture–gene coevolution, Tightness–looseness, Serotonin transporter gene, 5-HTTLPR, Moral justifiability

## Abstract

This research provides novel insights into the evolutionary basis of cultural norm development and maintenance. We yield evidence for a unique culture–gene coevolutionary model between ecological threat, allelic frequency of the serotonin transporter polymorphism (5-HTTLPR), cultural tightness–looseness—the strength of norms and tolerance for deviance from norms—and moral justifiability. As hypothesized, the results across 21 nations show that: (a) propensity for ecological threat correlates with short (S) allele frequency in the 5-HTTLPR, (b) allelic frequency in the 5-HTTLPR and vulnerability to ecological threat both correlate with cultural tightness–looseness, (c) susceptibility to ecological threat predicts tightness–looseness via the mediation of S allele carriers, and (d) frequency of S allele carriers predicts justifiability of morally relevant behavior via tightness–looseness. This research highlights the importance of studying the interplay between environmental, genetic, and cultural factors underlying contemporary differences in social behavior and presents an empirical framework for future research.

**Electronic supplementary material:**

The online version of this article (doi:10.1007/s40167-013-0009-x) contains supplementary material, which is available to authorized users.

## Introduction

**Fig. 1:**
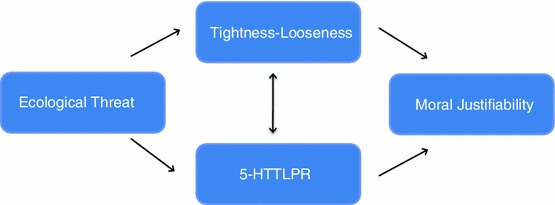
This culture–gene coevolutionary model depicts the predicted relationships between ecological threat, cultural tightness–looseness, the 5-HTTLPR polymorphism of the serotonin transporter gene, and moral justifiability

Judgments of whether morally contentious behaviors are permissible vary across the globe depending on the cultural norms and values of one’s society, yet little research has been done to explain such variation. In order to understand why humans can have such divergent perspectives on fundamental topics such as morality, we draw on culture–gene coevolutionary theory, which asserts that human behavior is influenced by two complementary and interacting processes: genetic and cultural selection (Boyd and Richerson 1985; Cavalli-Sforza 1981; Chiao and Blizinsky 2010; Lumsden and Wilson 1981). Within this theoretical framework, cultural norms and values, much like biological traits, are adaptive and may have emerged along side specific genetic variants in response to environmental pressures to produce and maintain advantageous behavior (Cheon et al. 2013; Chiao et al. 2013). This theory emphasizes that behavior is produced by a combination of factors including ecological pressures, culture, and genes. Our framework suggests that threats in the environment will affect both cultural selection and genetic selection, which in turn influence one another, and subsequently shape behavior and attitudes (Fig. 1).

The current study tests a novel example of culture–gene coevolutionary theory regarding the influences among tightness–looseness (TL), ecological threat, and allelic variants of the serotonin transporter linked polymorphic region (5-HTTLPR) in producing justification of moral behavior. Recent research highlights vast cultural differences among modern nations in the strength of social norms and tolerance of deviant behavior—the core components distinguishing tight and loose societies (Gelfand et al. 2011). Social norms and behavioral tolerance are represented in societal institutions and practices, and they are reflected in everyday social situations. For example, Gelfand et al. (2011) demonstrated that tight cultures (e.g. India, Singapore, Turkey, Japan) have higher situational constraint by which they have a more restricted range of appropriate behavior in everyday situations, compared to loose cultures (e.g. Estonia, Hungary, Israel, Netherlands).

Not only does TL influence norm enforcement, but individual psychological processes, such as self-regulation, also attune to strength of social norms. Individuals in tight (T) cultures, compared to loose (L) cultures, exhibit more cautious and dutiful behavior, higher self-regulatory strength, higher self-monitoring, and a greater need for structure (Gelfand et al. 2011). TL is related to, but distinct from, other well studied aspects of culture, such as individualism–collectivism (IND–COL). While TL primarily pertains to norm enforcement, IND–COL refers to the degree to which individuals feel strong ties to their ingroup. Gelfand et al. (2011) have shown that IND–COL is moderately and negatively correlated with TL.

TL is theorized to have evolved as a cultural adaptation to buffer against the presence of a broad array of biological, environmental, and human-made societal threats that vary across geographic regions (Gelfand et al. 2011). In essence, tight cultural norms are created and maintained to encourage social coordination that facilitates member survival in the face of frequent ecological threats. This structured coordination can help to reduce potential risks encountered by populations living in regions with higher population density, scarcity of resources, increased prevalence of natural disasters or territorial threats, and heightened transmission of disease via person-to-person contact (Gelfand et al. 2011). By contrast, nations with fewer ecological threats require less social order and coordination, leading to a looser and less stringent set of societal standards. Gelfand and colleagues' (2011) research from 33 nations illustrated a link between countries with greater ecological threats and nations with increased cultural tightness, even when controlling for economic indices, such as per capita GNP. Although TL has been shown to correlate with ecological threat, whether or not TL also has a genetic basis has yet to be determined.

We posit that genetic selection may also be influenced by ecological threat and play a role in shaping cultural selection of TL across geography. In other words, there may be genes that mediate the relationship between environmental pressure and TL. Despite strong evidence of geographic variability in TL across nations, little is known about what specific genes may underlie variation in cultural TL. Here we hypothesize that one specific gene, the serotonin transporter gene (SLC6A4) is likely to influence the cultural selection of TL. SLC6A4 contributes to regulation of serotonergic neurotransmission at the synapse (Canli and Lesch 2007; Lesch et al. 1996). This gene contains a promoter length polymorphic region, known as 5-HTTLPR, with two primary allelic variations- short (S) and long (L), corresponding to the length of the degenerate repeat. The two alleles differ in their transcriptional efficiency; the S allele is associated with reduced protein expression due to lower transcriptional efficiency (Lesch et al. 1996).

Prior research has shown that the S allele of the 5-HTTLPR length polymorphism is associated with increased negative emotion (Sen et al. 2004), increased harm avoidance (Munafo et al. 2005), enhanced fear acquisition (Lonsdorf et al. 2009), heightened attentional bias to negative information (Munafo et al. 2009), and augmented susceptibility to depression when faced with environmental risk factors such as stressful life events (Caspi et al. 2003; Taylor et al. 2006; Uher and McGuffin 2008), particularly in Western populations (Chiao and Blizinsky 2010). Given that the S allele of the 5-HTTLPR polymorphism is associated with an attentional bias to negative information, increased harm avoidance, and sensitivity to threat, it is plausible that S allele carriers are more adept at detecting environmental perils and successfully avoiding them due to heightened vigilance. Recent research has analogously demonstrated that countries with particular forms of high ecological threat, such as low disposable income spent on food and greater disease prevalence, also have higher allelic frequencies of S allele carriers and stronger social hierarchies (Fischer 2013). The author suggests that social hierarchies have helped societies at risk for clinical symptoms in threatening environments avoid dysfunction. Our present hypotheses are similar in the sense that they examine group-level evolutionary processes. Nonetheless, we examine a conceptually distinct cultural dimension (TL), and we suggest an example of the kind of social attitude that such an environment–gene–culture nexus can influence.

We posit that higher frequencies of S allele carriers persist in regions of the world prone to ecological hazards because heightened threat sensitivity may be adaptive in such regions (Chiao and Blizinsky 2010). Tighter cultural norms may have developed in these regions to increase social discipline in order to coordinate action and enhance safety within a population genetically at risk for anxiety. East Asian countries such as China and Japan have been shown to have tighter cultural norms (Gelfand et al. 2011), and these countries also have a greater proportion of S allele carriers (Gelernter et al. 1997; Nakamura et al. 1997).

Additionally, we examine the influence of both S allele frequency and TL on human sociality, specifically judgments on the justifiability of morally relevant behavior (Fig. 1). Morality is universal yet culturally variable; prior research has shown that judgments of moral justifiability are influenced by both cultural (Haidt and Joseph 2006; Rai and Fiske 2011) and genetic factors (Crockett et al. 2008, 2010; Marsh et al. 2011). Previous work has proposed that morality may be influenced by cultural evolution as much as genetic evolution (Haidt 2007), and we aim to highlight how these forces have worked together to produce global variation in moral judgments.

Variability in moral justifiability is associated with cultural TL (Gelfand et al. 2011). We suggest that the moral domain of any culture is rooted in the intolerance of particular forms of norm violation. In cultures where there are stricter norms, people should be much less tolerant of social deviations from normative behavior, especially for moral issues (e.g. divorce, prostitution, euthanasia); subsequently, people in such strict societies should judge these deviations from the norm as less justifiable. Indeed, as shown in Gelfand and colleagues' (2011) research, tight cultures, compared to loose cultures, have lower ratings of moral justifiability across an array of norm violating behaviors.

In addition to the influence of cultural variation, previous research suggests that moral justifiability also varies as a function of serotonergic activity. For instance, compared to people carrying two copies of the L allele, S allele carriers of the 5-HTTLPR have greater reluctance to endorse utilitarian actions resulting in foreseen harm to an innocent individual (Marsh et al. 2011). Similarly, when serotonin in healthy participants is enhanced with an SSRI, participants accept unfair offers in the ultimatum game to avoid harming their partner as well as judge harmful actions as forbidden when the harms were emotionally salient (Crockett et al. 2010). Hence, greater serotonin accumulation at the synapse enhances aversive emotional reactions to harm that may underlie avoidance of immoral behavior. We predict that in regions of the world with higher frequencies of S allele carriers, there is likely to be greater aversion toward harmful and morally questionable behaviors.

From the perspective of culture–gene coevolutionary theory, our hypotheses for the relationships between the discussed variables are twofold. Firstly, we suggest that TL and S allele variability are related to one another due to the influence of historical ecological threats. We hypothesize that ecological and human made threats increase the selection for S allele carriers, as these individuals are more likely to detect such environmental threats and avoid them. At the same time, because of the heightened sensitivity to threats, S allele individuals are also more inclined to develop strong norms that help them to coordinate actions to deal with these threats (Hypothesis 1). Hence, we hypothesize that increased vulnerability to ecological and human made threat will predict increased strength in social norms via heightened frequency of S allele carriers across nations. Secondly, we hypothesize that cultures with more S allele carriers are less likely to accept morally questionable behaviors because of their increased aversive emotional reactions to harm and unfairness, and this relationship should be explained by cultural variations in the strength of social norms. Put differently, cultures with more S allele carriers create stronger norms to foster social order and coordination and therefore challenges to the moral order are also not tolerated due to the strong constraints in the cultural context. Therefore, we predict that, across nations, cultural reactions to morally contentious behavior are indirectly affected by genetic adaptations (i.e. S allele) to the vulnerability of ecological threats through the strength of cultural norms (Hypothesis 2). Specifically, we hypothesize that greater S allele frequency will decrease judgments of justifiability of morally relevant, norm violating behavior via increased strength in social norms across nations.

## Materials and methods

To understand the relationships between ecological threat, TL, S allele frequency, and moral justifiability at a cross-national level, we integrated data collected across the globe to test our culture–gene coevolutionary hypothesis. We used published data on allelic frequency of the 5-HTTLPR length polymorphism from 50,135 individuals in 29 countries from 124 peer-reviewed publications collected between 1998 and 2008 (Chiao and Blizinsky 2010; Supplementary Table 1). Cultural TL scores were gathered from published data on 6,823 individuals across 33 nations collected between 2000 and 2003 (Gelfand et al. 2011; Supplementary Table 1). Higher TL scores indicate greater cultural tightness (i.e. strong social norms and low tolerance for deviance). Our analysis was performed on data from the 21 countries that overlapped between these datasets (Australia, Austria, Brazil, China, Estonia, France, Germany, Hungary, India, Israel, Italy, Japan, Mexico, Netherlands, New Zealand, Poland, Singapore, Spain, Turkey, United Kingdom, United States). Thus, our primary unit of analysis was geographical region defined by national boundaries. Several studies have demonstrated that geopolitical regions are sound proxies for cultural societies (Fincher et al. 2008; Schwartz 2004). To verify this, we also analyzed our data organized using Gupta and colleagues' (2002) ten distinct cultural clusters as the unit of analysis.

The composite ecological threat variable was comprised of standardized values of: (a) historical population density in 1500 AD accounting for national boundary shifts, (b) food deprivation as measured by the difference between the minimum dietary energy necessary and the average dietary energy intake within the undernourished population, (c) years of life lost to communicable disease measured by the frequency of premature deaths due to disease, (d) national vulnerability to natural disasters, and (e) prevalence of historical territorial conflicts between 1918 and 2001 (internal reliability of the composite variable as measured by Cronbach’s *a* = 0.93 as reported in the supplementary materials of Gelfand et al. 2011; Supplementary Table 1). Factor analysis demonstrated that these variables loaded onto one factor accounting for 79.63 % of total variance, with factor loadings ranging from 0.71 to 0.97. Data on population density in 1500 AD were not available for all countries, so for these countries Gelfand et al. (2011) used the average of the other threat values to compute the composite.^1^ These variables were selected primarily for theoretical reasons, because they cover a wide range of historical threats (resource scarcity, ecological threat, and human-made threat). For example, we operationalized resource scarcity with food deprivation, we operationalized ecological threat with vulnerability to disaster and communicable diseases, and we operationalized human-made threats with territorial conflict and population density. We argue that using an ecological threat variable that encompasses several historical forms of ecological threat is most effective for full representation of negative environmental influences, and this composite strengthens validity for measuring historical ecological threat predictive of TL (Gelfand et al. 2011). Data analyses were also conducted with historical and contemporary pathogen prevalence as reported in previous research (Chiao and Blizinsky 2010). The predictive value of these variables and mediation analyses were non-significant using pathogen prevalence, likely due to the fact that TL represents norm enforcement to facilitate social coordination when facing a variety of threats, beyond pathogen prevalence alone.

To account for potential economic factors, we incorporated information from all 21 countries on gross domestic product (GDP) and the GINI index, which represents inequality in income distribution. Given the prior demonstration of the relation between S allele frequency and cultural values of individualism–collectivism (IND–COL) referring to the degree to which individuals feel strong ties to their ingroup, we also included IND–COL as well as other cultural values (e.g. Power Distance) in various regression models to demonstrate the unique relationship between TL and S allele frequency (Table 4).

Cross-national data on the justifiability of morally relevant behavior were gathered from the 1995 World Value Survey for 19 of the 21 nations analyzed in the present study. The World Value Survey used items from the Morally Debatable Behaviors Scale (MDBS; Harding and Phillips 1986), which was developed to measure the extent to which contestable behaviors are viewed as justifiable as part of a larger cultural value study in Europe in the mid-1980s. All justifiability scores were the standardized averages reported in the TL work of Gelfand et al. (2011)^2^ (Supplementary Table 1). Moral justifiability was measured on a scale of one (never justifiable) to ten (always justifiable), and individuals rated whether they believed the following behaviors to be justified: “claiming government benefits to which you are not entitled”, “avoiding a fare on public transit”, “cheating on taxes if you have a chance”, “homosexuality”, “prostitution”, “abortion”, “divorce”, “euthanasia—ending the life of the incurably sick”, and “suicide” (Cronbach’s *a* = 0.73; World Values Survey 1995). Although these items are not necessarily moral in nature, the nine behaviors represent various types of deviations from normative standards. In order to make judgments on the justifiability of these behaviors, one needs to evoke some moral convention or intuition; otherwise, it is difficult to decide whether the behavior at hand is right or wrong/justifiable or not justifiable.

Recent research on the MDBS has demonstrated that there are two individual-level factors within this scale (personal-sexual, illegal-dishonesty) that relate to other cultural values (Minkov et al. 2012; Vauclair and Fischer 2011). These two levels have higher internal reliability when separated (Cronbach’s *a* for personal-sexual dimension = 0.88; Cronbach’s *a* for illegal-dishonesty dimension = 0.87). However, S allele frequency theoretically should predict lower moral justifiability due to increased sensitivity to both harm and fairness across domains, so the analyses in this study are conducted with both levels included to operationalize moral justifiability.^3^

We used standard multiple regression and mediation analytic techniques (Baron and Kenny 1986; Preacher and Hayes 2004) to examine the relationship between ecological threat, 5-HTTLPR, cultural TL, and moral justifiability. Additionally, we combined these four variables into a structural equation model to avoid possible error of multiple statistical analyses.

## Results

To test our culture–gene coevolutionary theory, we will demonstrate (a) correlations among all variables, (b) mediation model supporting Hypothesis 1, and (c) mediation model and structural equation model supporting Hypothesis 2. Due to our strong directional hypotheses based on previous work (Gelfand et al. 2011), we used one-tailed statistical testing for all analyses.

**Fig. 2:**
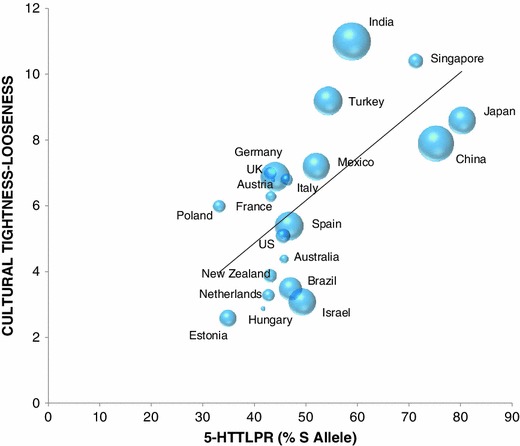
Mean values of cultural tightness and S allele frequency of the 5-HTTLPR polymorphism of the serotonin transporter gene across 21 countries. **The greater the bubble size associated with each country represents greater ecological threat in that nation**

First, we found evidence of an association between allelic frequency in the length polymorphism (5-HTTLPR) of the serotonin transporter gene and TL. As hypothesized, cultural TL was significantly, positively correlated with S allele frequency across nations [*r*(21) = 0.65, *p* = 0.001, Table 1; Fig. 2].^4^ This correlation remained significant when Gupta et al. (2002) ten distinct cultural clusters were substituted as the unit of analysis [*r*(8) = 0.77, *p* = 0.01].

**Table 1:** Correlation matrix of primary variables of interest across 21 nations

	Tightness–looseness	Ecological threat	S allele frequency	Moral justifiability
Tightness–looseness				
Correlation	_			
*p* value				
Covariance				
Ecological threat				
Correlation	0.479*	_		
*p* value	0.014			
Covariance	0.952			
S allele frequency				
Correlation	0.650**	0.559**	_	
*p* value	0.001	0.004		
Covariance	19.642	5.568		
Moral justifiability				
Correlation	−0.748**	−0.427*	−0.492*	_
*p* value	<0.001	0.034	0.016	
Covariance	−0.952	−0.182	−3.257	

One-tailed correlations, (* *p* < 0.05; ** *p* < 0.01)

Consistent with our hypothesis that global variation of S allele frequency is related to ecological threat, there was a significant, positive correlation between measures of ecological threat and S allele frequency [*r*(21) = 0.56, *p* = 0.004, Fig. 2]. Even with Gupta and colleagues' (2002) ten cultural clusters, the correlation between ecological threat and S allele frequency remained significant [*r*(8) = 0.65, *p* = 0.04]. Similar to the results of Gelfand et al. (2011), we found that ecological threat significantly correlated with TL, using nations [*r*(21) = 0.48, *p* = 0.01] of Gupta and Hanges’ cultural clusters [*r*(8) = 0.89, *p* = 0.002] as the unit of analysis.

**Fig. 3:**
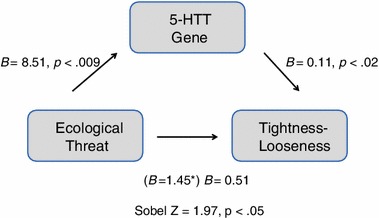
Mediation analyses illustrating relationships between historical ecological threat, S allele frequency of the 5-HTTLPR length polymorphism, and cultural tightness–looseness

According to culture–gene coevolutionary theory, we hypothesized that the effect of ecological threats on TL is mediated by S allele frequency across cultures (Hypothesis 1). Indeed, mediation regression analyses using a bootstrapping approach supported this hypothesis. The direct effect of ecological threat on TL across the 21 nations (*B* = 8.51, *p* < 0.009) decreased significantly when cultural variation of S allele frequency was taken into account (Sobel test *Z* = 1.97, *p* < 0.05; Fig. 3; Baron and Kenny 1986; Preacher and Hayes 2004). These results suggest that ecological threat predicts TL due to increased frequency of the 5-HTTLPR S allele.

**Fig. 4:**
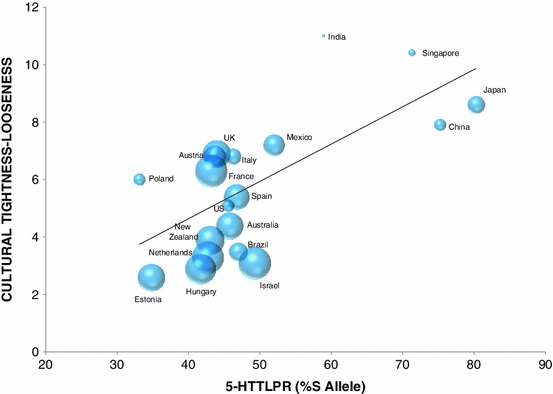
Mean values of cultural tightness and S allele frequency of the 5-HTTLPR polymorphism of the serotonin transporter gene across 19 countries. **The greater the bubble size associated with each country represents greater justifiability of morally relevant behavior in that nation**

Next, we tested whether S allele frequency is related to moral justifiability and whether this relationship can be explained by TL. Aligning with our hypotheses, there was a significant, negative correlation between S allele frequency and moral justifiability [*r*(19) = −0.43, *p* = 0.02, Fig. 4], as well as between TL and moral justifiability [*r*(19) = −0.75, *p* < 0.001, Fig. 4].

**Fig. 5:**
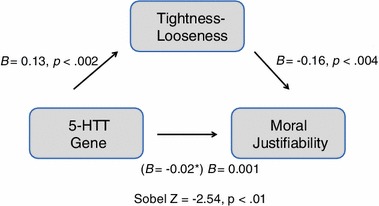
Mediation analyses illustrating relationships between S allele frequency of the 5-HTTLPR length polymorphism, cultural tightness–looseness, and mean ratings of moral justifiability

To test our prediction that the relationship between S allele frequency and moral justifiability is mediated by TL based on our novel culture–gene coevolutionary model (Hypothesis 2), we again used mediation analyses with bootstrapping. When TL was taken into account, the effect of S allele frequency on moral justifiability decreased significantly (*B*(19) = −0.02, *p* = 0.03 to *B*(19) = 0.001, *p* = 0.92; Sobel test *Z* = −2.54, *p* < 0.01; Fig. 5). Our results demonstrate for the first time that S allele frequency predicts decreased moral justifiability due to increased cultural tightness.

**Fig. 6:**
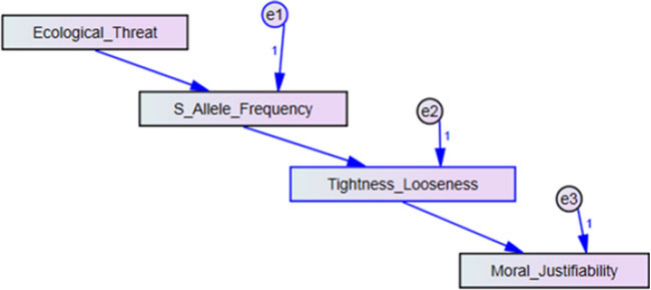
Structural equation model of ecological threat, S allele frequency, tightness–looseness, and moral justifiability across 19 nations using a bootstrapping approach. All available published data for each variable were included

To examine an overall model fit, the results supported our hypothesis by demonstrating pathways of ecological threat → S allele frequency → TL → moral justifiability. In order to avoid the risk of enhanced error due to multiple statistical analyses and to evaluate the degree to which our theoretically derived model fits observed data, we also created a structural equation model with standardized ecological threat serving as an exogenous variable and standardized versions of TL, S allele frequency, and moral justifiability serving as endogenous variables in Mplus and SPSS Amos (see Fig. 6).

Our results also show a significant mediation model between ecological threat and S allele frequency via TL. Specifically, when TL was taken into account, the effect of ecological threat on S allele frequency decreased significantly (*B*(21) = 8.51, *p* = 0.008 to *B*(21) = 4.90, *p* = 0.12; Sobel test *Z* = 1.76, *p* < 0.05. This reverse mediation model may help justify the coevolution argument between TL and S allele frequency, because it demonstrates that each variable has a predictive influence on the other. Thus, for a comparison model, we used the pathway of ecological threat → TL → S allele frequency → moral justifiability.

We used maximum likelihood techniques to determine the fit of our model, and results revealed an adequately strong fit (Chi squared = 1.099, *p* = 0.777, 3° of freedom; see Tables 1, 2, and 3). The comparison model (i.e. the model where TL and S allele frequency are reversed) fit less well (Chi squared = 13.805, *p* = 0.003, 3° of freedom). Our hypothesized model had a root mean square error of approximation <0.001, whereas the comparison model was 0.414; similarly, our model had a lower Akaike information criterion (AIC) = 271.116, compared to the comparison model, AIC = 283.822. The good fit of our structural equation model supports the hypothesized causal pathways among the variables of interest.

**Table 2:** Coefficients and standard errors for all estimated pathways

	Estimate	S.E.	P value
S allele frequency ← ecological threat	8.505	2.753	0.002
Tightness–looseness ← S allele frequency	0.130	0.033	<0.001
Moral justifiability ← Tightness–looseness	−0.156	0.032	<0.001

As hypothesized, there are significant pathways from ecological threat to S allele frequency, from S allele frequency to TL, and from TL to moral justifiability

**Table 3:** Means, standard deviations and sample sizes for all variables included in the structural equation model

	Mean	Std. deviation	*N*
Tightness–looseness	6.110	2.456	21
Ecological threat	0.062	0.809	21
S allele frequency	49.600	12.303	21
Moral justifiability	0.027	0.516	19

Given that the model shows that S allele frequency predicts TL, it is important to test whether such an effect may be explained by economic indices and related cultural variables; hence, we conducted a series of additional regressions. To examine whether the relationship between TL and S allele frequency was due to economic factors, we created a regression model with S allele frequency, GDP, and GINI index as predictors of TL [*R* ^2^ = 0.43, *F* (5,20) = 4.33, *p* = 0.02]. S allele frequency was predictive of TL above and beyond the influence of economic factors (Table 4). Similarly, we also ran a regression with GDP, GINI index, ecological threat, and S allele frequency as predictors of TL [*R* ^2^ = 0.45, *F* (4,20) = 3.31, *p* = 0.04]. Despite the interrelatedness of many of these variables, S allele frequency was the only significant predictor of TL (*β* = 0.58, *p* = 0.04, Table 4). When including economic factors of per capita GDP and GINI, ecological threat remained the only significant predictor of S allele frequency [*R* ^2^ = 0.51, *F* (3,20) = 5.83, *p* = 0.006].

**Table 4:**
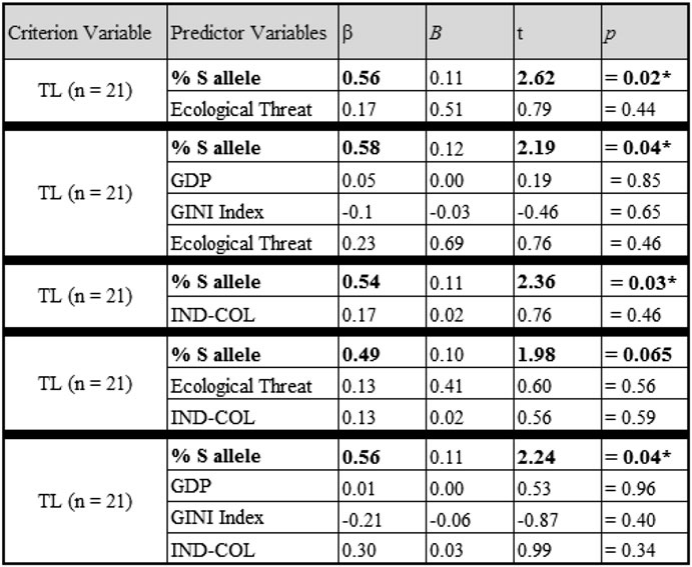
Results from multiple regression analyses examining the association between cultural values of tightness–looseness and allelic frequency of the polymorphic serotonin transporter gene across 21 nations

All available published data for each variable were included in the regression analyses (*β* standardized beta coefficients, *B* unstandardized beta coefficients * *p* < 0.05; ** *p* < 0.01)

Previous research has illustrated the significant relationship between S allele frequency and IND–COL^5^ (Chiao and Blizinsky 2010) as well as TL and IND–COL (Gelfand et al. 2011). To examine the uniqueness of the link between S allele frequency and TL, we ran a regression model that included allelic frequency of the serotonin transporter gene and IND–COL as predictors of TL [*R* ^2^ = 0.44, *F* (2,20) = 7.09, *p* = 0.005]. Consistent with prior demonstration that TL is a distinct cultural construct from IND–COL, we found that S allele frequency significantly predicts TL, even when controlling for IND–COL, [*β* = 0.54, *p* = 0.03, Table 4]. We ran an additional regression to test the uniqueness of the link between TL and S allele frequency above and beyond IND–COL when controlling for economic variables of GDP and Gini index [*R* ^2^ = 0.47, *F* (4,20) = 3.49, *p* = 0.03]. In this regression, only S allele frequency predicted TL [*β* = 0.56, *p* = 0.04, Table 4]. Similarly, we also ran a regression to test the uniqueness of the link between TL and S allele frequency above and beyond IND–COL when controlling for ecological threat [*R* ^2^ = 0.45, *F* (4,20) = 4.68, *p* = 0.02]. S allele frequency was the only predictive variable of TL, though this effect was marginal [*β* = 0.49, *p* = 0.065, Table 4]. Together, these three regressions demonstrate that there is a unique relationship between TL and S allele frequency above and beyond the cultural influence of IND–COL, even when controlling for ecological and economic indices.

## Discussion

A key mandate in cultural neuroscience is to understand how wide variation in behavior develops across the globe. The results from this study highlight the importance of both environmental and genetic factors in explaining cultural differences, as well as how variation in such cultural and genetic factors can produce social and moral attitudes across the globe. To our knowledge, this is the first work to present evidence of the culture–gene coevolution of social attitudes. Our findings suggest that global variation in TL is influenced by ecological threat, and this association is mediated by allelic variation in the serotonin transporter gene (5-HTTLPR). Additionally, we demonstrated that allelic variation in the serotonin transporter gene affects moral justifiability via mediation of cultural TL.

According to our results, regions faced with ecological threat have a higher frequency of individuals carrying the S allele, who have been shown to exhibit greater capacity in detecting threats in the environment (e.g. heightened attention to negative information, harm avoidance). Tight cultural norms may have emerged as adaptive mechanisms that attenuate the risks of both genetic vulnerability for anxiety and environmental threats through the heightened need for coordination for survival. This theory is supported by the results of a significant mediation model whereby the previously demonstrated link between ecological threat and TL becomes non-significant when 5-HTTLPR is included as a mediator. This mediation suggests that historical ecological threat led to the development of cultural norms of TL, due in part to S allele carriers’ vigilance toward threat.

The present culture–gene coevolutionary model is the first to empirically demonstrate how environmental pressures influence cultural variation via genes and how genes influence human social judgments via cultural variation. Moral permissibility has long been viewed as a product of cultural differences, and variation in TL is a robust covariate of moral justifiability (Gelfand et al. 2011). Accumulating research also suggests that morality is partially genetically driven by individual differences in serotonergic activity.

Our results suggest that global variation in moral justifiability is influenced by both genetic variation as well as cultural systems such as TL; however, the relation between S allele frequency and moral justifiability becomes non-significant when accounting for TL. This model is not significant when moral justifiability acts as the mediator between S allele frequency and TL, suggesting that allelic frequency in the serotonin transporter gene is predictive of moral justifiability via the influence of TL rather than allelic frequency predicting TL via judgments of moral justifiability.

Although our model supported a causal pathway from genes to culture, data collected over a much longer timeframe might also illustrate that TL influences genetic selection. According to culture–gene coevolution, in cultures where tight norms are strictly enforced, individuals carrying the S allele may be more successful at vigilantly following the norms, and ultimately would likely be selected for. Accordingly, cultural tightness may influence the allelic variation within the genetic pool across populations over time. Nonetheless, in the current study, our structural equation model suggests that our hypothesized causal pathway (ecological threat → S allele frequency → TL → moral justifiability) is stronger than the comparison model where S allele frequency and TL are reversed. Additionally, the comparison model does not have a strong impact on moral justifiability, but it may be useful in examining future outcome variables.

The present study is not without limitations. For example, we examined data from only 21 countries; hence it is plausible that our existing knowledge of cultural and genetic variation is limited. An additional drawback of this research is that because this data is primarily correlational, causal inferences cannot be determined. Our mediation analyses imply directionality, but future experimental work involving genotyping, behavioral priming of TL, and moral justifiability paradigms will valuably strengthen the understanding of these correlations.

In a recent response to Chiao and Blizinsky (2010), Eisenberg and Hayes (2011) cautioned that a major challenge of demonstrating culture–gene coevolution in human behavior is ensuring that allelic variation is due to natural selection, rather than neutral processes such as the founder effect or genetic drift. If this pattern were to emerge as a product of neutral selection, then it would entail that TL and allelic variation in the 5-HTTLPR correlate with one another across geographic regions by chance. It is highly unlikely that S allele frequency and TL are higher in regions with greater threat and lower in regions with less threat by coincidence.

Based on the theory of genetic drift, allelic frequencies become polarized over time due to inbreeding within a community that has a particular allelic content determined by chance. Because this pattern is upheld across diverse geographic regions, it is unlikely that it emerged due to genetic drift.

Similarly, if this pattern is due to the founder effect, this would entail that groups of S allele carriers happened to settle in regions with high ecological threat by chance. On the contrary, this pattern subsists across the world with ecologically threatening regions geographically dispersed; hence, it would be improbable for this global pattern to have emerged from groups of S allele carriers coincidentally migrating to regions with greater threat. Although improbable, some may argue that LL individuals migrated to areas with low ecological threat. Previous research has demonstrated that particular genotypes are more likely to migrate to certain regions, which would support the Founder Effect, yet no evidence has been shown for our gene of interest, the serotonin transporter gene (Chen et al. 1999, Matthews and Butler 2011). Future research on the serotonin transporter gene and the possibility of neutral selection would be useful.

Here we demonstrate that even in geographic regions with reduced ecological threat, the relationship between S allele frequency and TL remains significant. Excluding Asian countries with high S allele frequency and high ecological threat (Japan, China, and India), S allele frequency still predicts TL [*r*(18) = 0.64, *p* = 0.002, one-tailed]. This pattern elucidates the fact that allelic differences in the length polymorphism of the serotonin transporter gene vary in response to subtle differences in the degree of ecological threat, making it much more probable that this relationship is due to natural selective processes. Hence, we posit that the association between allelic frequency of the 5-HTTLPR polymorphism of the serotonin transporter gene and TL is due to natural selection via situational advantageousness. Individuals carrying the S allele may have an advantage over their less vigilant counterparts when facing ecological threats; thus, communities highly comprised of S allele carriers may more successfully attune to environmental threats and socially coordinate to avoid these threats in regions where they are particularly prevalent.

Our study provides novel insights into the coevolutionary influences on the creation and maintenance of cultural norms as well as the production of attitudes on morally contentious behavior. This research underscores the importance of studying cultural and genetic differences in empirical models that aim to understand variation in human behavior and attitudes. Future research would benefit from examining other specific genetic polymorphisms that may be associated with TL, as well as examining the environment-culture–gene associations that influence the psychological and neural processes underlying complex human behavior (Chiao et al. 2010, 2013).

In summary, the current findings demonstrate for the first time the significant relationship between TL and allelic variation in the serotonin transporter gene, as well as the interplay between these variables in predicting judgments of moral justifiability. This research highlights the importance of culture–gene coevolutionary theory in studying the predictive factors behind modern day differences in human social behavior and provides an empirical framework for future research.

## Electronic supplementary material

Below is the link to the electronic supplementary material.

Supplementary material 1 (PDF 65 kb)

## Footnotes

1 When population density in 1,500 is substituted with rural population density, the composite is nearly identical (Cronbach’s *a* = 0.97), indicating that the seemingly arbitrary date of 1,500 does not influence the composite variable.

2 Justifiability for morally relevant behavior was not available for Germany or Turkey.

3 Aligned with our theoretical rationale for the link between S allele frequency and moral justifiability, S allele frequency correlates with the composite of these levels (personal-sexual and illegal-dishonesty), rather than with the two levels independently.

4 If we divide the dataset into Eastern countries and Western countries, our correlations no longer remain significant. However, this is likely due to small sample size (for Eastern countries, *n* = 4).

5 In this dataset, S allele frequency is correlated with several cultural variables in addition to TL including IND–COL, power distance, and long term orientation. When these three cultural variables are included in a regression with S allele frequency as predictors of TL [*R* ^2^ = 0.55, *F* (4,20) = 4.782, *p* = 0.01], only S allele frequency is a significant predictor of TL [*β* = 0.71, *p* = 0.02].
