# Supplementary material for: The role of culture–gene coevolution in morality judgment: examining the interplay between tightness–looseness and allelic variation of the serotonin transporter gene
Source: Cult Brain. 2013 Aug 29;1(2):100–17. doi: 10.1007/s40167-013-0009-x (PMC3880222; doi:10.1007/s40167-013-0009-x)
Supplement: Supplementary file 1 — Supplementary material 1 (PDF 65 kb) [file 40167_2013_9_MOESM1_ESM.pdf]

Supplementary Table 1.

	5-HTTLPR				IND-COL	TL	Economic		Pathogen Prevalence		EcoThreat	Moral
**Nation**	**No. of Studies**	**N**	**% S**	**% L**	**SCSI_rev**	**TL**	**GDP**	**Gini**	**Pathogen Historical**	**Pathogen Contemp.**	**Composite**	**Justifi-ability**
Argentina	2	2012	51.04	48.96	54		13100	49	-0.07	37		
Australia	4	1758	45.91	54.09	10	4.4	37300	30.5	-0.2	27	-0.68	0.31
Austria	2	416	43.65	56.35	45	6.8	39300	26	-0.72	26	-0.82	0.02
Belgium						5.6						0.47
Brazil	6	1747	46.96	53.04	62	3.5	9500	56.7	1.02	45	0.22	-0.19
Denmark	1	1369	40.80	59.20	26		37200	24	-0.93	25		
Estonia	2	808	34.81	65.19	40	2.6	21800	34	-0.55	25	-0.27	0.35
Finland	2	4269	42.45	57.55	37		36000	26	-0.8	25		
France	8	2665	43.18	56.82	29	6.3	32600	28	-0.51	29	-0.61	0.7
Germany	12	4105	43.03	56.97	33	7.0	34100	28	-0.93	24	-0.53	
Greece						3.9						0.62
Hong Kong					75	6.3	42000	53.3	0.32	25		-0.13
Hungary	4	1067	41.71	58.29	20	2.9	19300	28	-0.93	28	-0.78	0.58
Iceland						6.4						0.25
India	3	1007	58.85	41.15	52	11.0	2600	36.8	0.9	39	1.95	-1.14
Indonesia					86		3600	36.3	0.72	37		
Israel	8	2561	49.26	50.74	46	3.1	26600	38.6	0.6	28	0.6	0.71
Italy	4	876	48.54	51.46	24	6.8	30900	33	0.22	26	-0.53	-0.29
Japan	5	1176	80.25	19.75	54	8.6	33500	38.1	0.51	28	0.62	-0.24
Korea	5	931	79.45	20.55	82	10.0	25000	35.1	0	32		-0.44
Malaysia						11.8						0.28
Mexico	3	380	51.96	48.04	70	7.2	12400	50.9	0.26	37	0.6	-0.04
Netherlands	1	989	42.72	57.28		3.3	39000	30.9	-0.93	24	-0.53	0.67
New Zealand	1	847	43.03	56.97	21	3.9	27200	36.2	-1.16	23	-0.47	0.43
Nigeria							2100	43.7	1.28	47		
Norway						9.5						0.27
Pakistan						12.3						-1.86
Poland	4	696	36.96	63.04	40	6.0	16200	36	-0.8	27	-0.52	-0.44
PR China	3	1896	75.20	24.80	80	7.9	5400	47	1	37	1.62	-0.44
Portugal						7.8						-0.43
Puerto Rico							18400	61	0.16	27		
Russia	4	1370	43.91	56.09	61		14800	41.3	-0.42	28		
S. Africa	2	753	27.79	72.21	35		9700	65	0.09	36		
Singapore	2	629	71.24	28.76	80	10.4	49900	52.2	0.36	26	-0.4	-0.63
Slovenia	1	468	42.52	57.48			28000	24	-0.8	27		
Spain	8	3152	46.75	53.25	49	5.4	33600	32	0.03	31	0.74	0.2
Sweden	3	752	43.63	56.37	29		37500	23	-0.93	25		
Taiwan	1	192	70.57	29.43	83		30100	32.6	0.34	29		
Turkey	6	1194	54.29	45.71	63	9.2	12000	43.6	0.15	39	0.74	
Ukraine						1.6						0.31
UK	3	5888	43.98	56.02	11	6.9	35000	34	-0.96	26	0.79	0.37
USA	14	4162	44.53	55.47	9	5.1	45800	45	-0.86	29	-0.42	-0.43
Venezuela						3.7						-0.89

Note: Red highlight indicates inclusion in regression and mediation analyses
